# Surgical Management of Temporal Lobe Epilepsy Secondary to Epidermoid Cysts: A Case Report With Review of the Literature

**DOI:** 10.7759/cureus.45360

**Published:** 2023-09-16

**Authors:** Carlos Salvador Ovalle Torres, Ibrahim E Efe, Manuel de Jesus Encarnacion Ramirez, Eduardo Diaz Juarez, Angel Ruano Calderon, Renat Nurmukhametov, Alvaro Campero, Issael Jesus Ramirez Pena, Nicola Montemurro

**Affiliations:** 1 Neurosurgery, National Autonomous University of Mexico, General Hospital, Mexico, MEX; 2 Neurosurgery, Charité - Universitätsmedizin Berlin, Berlin, DEU; 3 Neurosurgery, Peoples' Friendship University of Russia, Moscow, RUS; 4 Neurosurgery, National Autonomous University of Mexico, Mexico, MEX; 5 Neurosurgery, Hospital Ángel C. Padilla, San Miguel de Tucumán, ARG; 6 Neurooncology, Royal Melbourne Hospital, Melbourne, AUS; 7 Neurosurgery, Azienda Ospedaliero Universitaria Pisana (AOUP), Pisa, ITA

**Keywords:** cerebellopontine angle, epidermoid cyst, lesionectomy, tumor-related epilepsy, temporal lobe epilepsy

## Abstract

Epidermoid cysts represent roughly 1% of all intracranial tumors. They are frequently located in the cerebellopontine angle but rarely extend to the supratentorial brain. Epilepsy is an extremely uncommon manifestation of this neoplasm. We suggest the surgical management of a 35-year-old male who presented with a six-month history of intractable temporal lobe epilepsy. His seizures were characterized by a focal onset in the form of déjà vu experiences, followed by a secondarily generalized tonic-clonic seizure. Imaging revealed a heterogeneous cystic mass in the right cerebellopontine angle, extending supratentorially causing a mass effect on the mesial temporal region. Gross total resection was achieved through a combined subtemporal-retrosigmoid approach. Histopathology revealed an epidermoid cyst. The patient was entirely seizure-free at the three-month follow-up. Epidermoid cysts may present with epileptic seizures. Seizure freedom can be achieved with surgical management in most cases. The patient’s symptoms, imaging findings, and epileptogenic focus must be considered to select the appropriate surgical strategy.

## Introduction

Intracranial epidermoid cysts are rare, benign, and slow-growing lesions, representing 0.3-1.8% of all intracranial tumors [1]. They are predominantly formed between the third and fifth week of gestation due to the inclusion of ectodermal elements during neural tube closure [2]. However, they typically remain unnoticed until the third to fifth decade of life. They are found in the cerebellopontine (CP) angle in 40% of cases [1]. Epidermoid cysts present with a thin capsule of stratified keratinized squamous epithelium and fatty acids, giving these lesions a pearlescent appearance intraoperatively. They rarely transform into malignant lesions during the course of the disease [3-5]. Patients can suffer from a wide range of symptoms, including hearing loss, dizziness, gait disturbance, and headache [6-9]. In magnetic resonance imaging (MRI), epidermoid cysts present as hypointense lesions in T1-weighted and hyperintense in T2-weighted and diffusion-weighted imaging [6,7].

Epilepsy, however, is an extremely uncommon manifestation rarely reported in the literature. Gross total resection of the cyst along with its capsule should be attempted. However, these tumors may be strongly attached to the brain parenchyma, rendering complete resection difficult. We here present the rare case of epidermoid cyst-related drug-refractory temporal lobe epilepsy in a male patient and provide an overview of all reports of epilepsy secondary to epidermoid cysts found in the literature [5,8].

## Case presentation

A 35-year-old man was referred after a neurological consultation to our neurosurgical department with a six-month history of drug-refractory epilepsy. He had a long history of substance abuse. His past medical history was otherwise uneventful. The patient’s seizures were characterized by déjà vu experiences and feelings of fear, suggestive of focal temporal lobe epilepsy. Seizures occurred one to two times per month. After focal onset, the seizure was secondarily generalized into a bilateral tonic-clonic seizure with three to four minutes in length. The postictal phase was typically characterized by a 30-min episode of prosopagnosia and disorientation to time and place. The patient further complained about occasional diffuse headaches within the last six months. He had no focal neurological deficits and achieved the maximum score of 30 in the mini-mental state status (MMSS).

**Figure 1:**
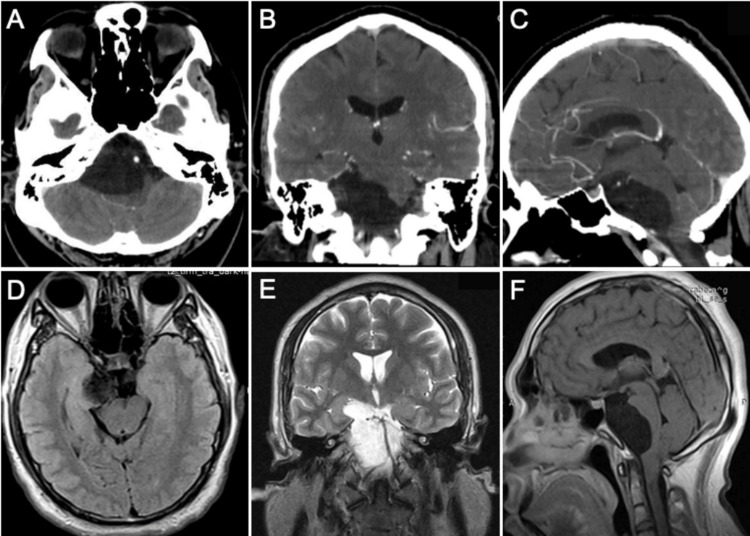
Preoperative MRI. Contrast-enhanced CT showing a cystic lesion displacing the basilar artery to the left side on an axial image (A) and compressing the right uncus on a coronal image (B). The mass effect on the brain stem can be appreciated in the sagittal image (C). The lesion appeared hypointense on T1-weighted MRI (D) and hyperintense on T2-weighted MRI, extending supratentorially, causing compression of the mesial temporal lobe (E). On the sagittal fluid-attenuated inversion recovery (FLAIR) MRI, the cystic content appears heterogeneous with no perilesional edema (F).

In our department, the patient underwent a computer tomography (CT) showing a hypointense well-delimited cystic mass in the right CP angle (Figure 1).

**Figure 2:**
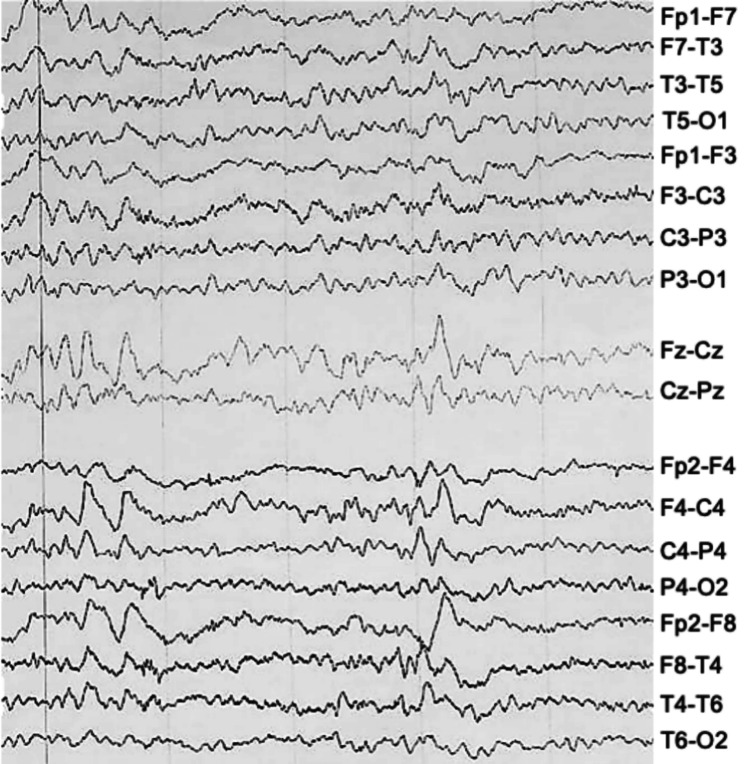
Sleep-deprived EEG. Sleep-deprived EEG showing epileptiform activity with sharp waves in the theta and delta ranges, predominantly in the frontal and temporal regions, suggesting cortico-subcortical dysfunction.

The cystic content appeared heterogeneous in MRI. The mass measured roughly 6 x 2 x 5 cm and extended supratentorially compressing the right-sided mesial temporal region. It occupied the right CP and interpeduncular cisterns as well as the entire prepontine cistern, extending into the foramen magnum. The basilar artery was displaced contralaterally. A sleep-deprived electroencephalogram (EEG) showed epileptiform activity in the frontal and temporal regions (Figure 2).

**Figure 3:**
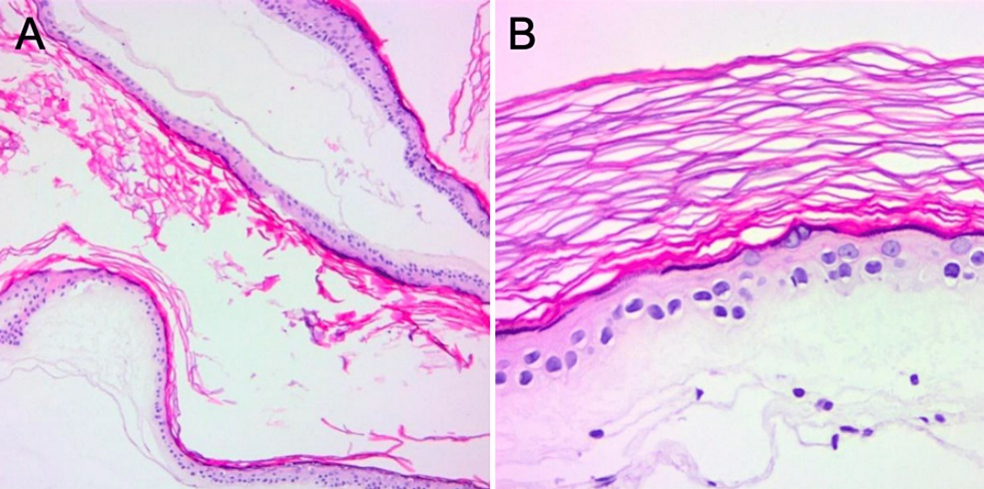
HE staining. Stratified squamous epithelium delimiting the cystic mass and lamellar keratin fibers characteristic for an epidermoid cyst (A) (HE staining, 100x). Keratohyaline granules and lamellar keratin fibers (B) (HE staining, 400x).

We hypothesized the patient’s epilepsy to originate from the mass effect of the cystic lesion and decided to perform a microsurgical resection with intracranial EEG, through a combined subtemporal-retrosigmoid approach, to expose the whole lesion. We started with the retrosigmoid craniotomy to visualize the most caudal part of the mass. We drained cerebrospinal fluid from the cisterna magna to achieve cerebellar relaxation and to access the CP angle. The cyst wall ruptured when trying to prepare a resection plane, releasing yellowish-crystalline fluid. We immediately aspirated the intracapsular content and could significantly debulk the infratentorial part of the lesion. We then irrigated copiously to aspirate any remaining cyst content from the prepontine cistern. As the cyst capsule was adherent, particularly to cranial nerves six, seven, and eight, thorough visualization of the lower cranial nerves was paramount before meticulous sharp dissection of the capsule residuals. We then moved on to the temporal craniotomy. Dural opening was performed with a T-shaped durotomy. The temporal lobe was retracted to visualize the optico-carotid and suprachiasmatic cisterns as well as the interpeduncular and quadrigeminal cisterns. The remaining cystic content was aspirated and the residual capsule was sharply dissected off the third and fourth cranial nerves. The mesial temporal cortex had been fully decompressed after successful debulking of the mass. Radical detachment of the capsule from the cortical surface was thus not attempted. The surgery was completed with no major blood loss and the postoperative course was uneventful. Histopathological examination confirmed the diagnosis of epidermoid cyst (Figure 3).

**Figure 4:**
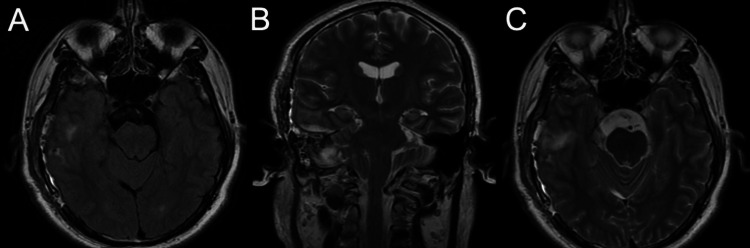
Postoperative MRI. Follow-up MRI at three months post-surgery showing a fully decompressed mesial temporal lobe and a minimal residual of the capsule in an axial FLAIR image (A), coronal T2-weighted image (B), and axial T2-weighted image (C).

The patient was discharged in good condition one-week post-surgery, after surgical mobilization and rehabilitation. In the follow-up MRI at three months post-surgery, no evidence of growth of the capsular remnant was seen (Figure 4). The patient was entirely seizure-free. A low-dose (500 mg x 2) levetiracetam was continued for 12 months.

## Discussion

Temporal lobe epilepsy secondary to epidermoid cysts is a rare and poorly understood phenomenon. Data are scarce, and there is no guideline on which surgical strategy to opt for. However, previous case reports have shown seizure-free after radical surgical management of these lesions (Table 1) [1,10-15].

**Table 1:** Previous reports of temporal lobe epilepsy secondary to epidermoid cyst.

Study	Age (y)/Sex	Clinical presentation	Etiology	Treatment	Outcome
Trindade et al. [14]	59/M	Sudden onset bad smell sensations, secondarily generalized tonic-clonic convulsions	Large subtemporal epidermoid cyst affecting anterior, middle, and posterior fossa and invasion of the choroidal fissure	Lesionectomy through a pretemporal craniotomy and combined transsylvian and subtemporal approach	Seizure-free at 1-year follow-up
Wong et al. [15]	33/F	6 months history of déjà vu episodes, followed by sensory overload, distortion of immediate surroundings, sensation of “head being trapped inside a fishbowl”, nominal memory difficulties, crying after a seizure event	Large epidermoid tumor extending from the left CP angle to the medial aspect of the left temporal lobe with invagination and displacement of the left hippocampus	Lamotrigine 100 mg 2x/day, clobazam 10 mg 1x/day, subsequent cytoreductive surgery	Medication had modest effect. Patient became seizure-free after surgery.
Hiraishi et al. [11]	31/F	Daily strange sensations like “flying into another world” since childhood, first generalized tonic-clonic seizure at 30 years of age	Epidermoid cyst involving the right basal cistern and inferior horn of the lateral ventricle	Anterior temporal lobectomy and amygdalo-hippocampectomy	Seizure-free at 40-months follow-up
Hanft et al. [10]	19/F	Secondarily generalized tonic-clonic seizure	Right superior temporal lobe epidermoid cyst	Lesionectomy through awake right temporal craniotomy	Seizure-free at 5-year follow-up
Hanft et al. [10]	71/M	Long history of seizures, worsening headache	Left temporal lobe epidermoid cyst infiltrating middle and inferior temporal gyri	Lesionectomy through left temporal craniotomy	Postoperative remission of seizures
Sharifi et al. [12]	19/F	Automatisms in upper extremities and face, occasional secondary tonic-clonic generalization since the age of 10	Left temporal epidermoid cyst	Lesionectomy through a left pterional trans-sylvian trans-ventricular approach	Seizure-free at 1-year follow-up
Tanriover et al. [1]	25/F	Complex visual hallucinations	Right temporobasal epidermoid tumor on the basal surface of the temporal lobe, within the collateral sulcus	Right-sided anterior temporal lobectomy and microsurgical removal of the epidermoid tumor and mesial temporal structures	Seizure-free after surgery
Taniguchi et al. [13]	NA	Epileptic laughter	Deep right temporal epidermoid cyst	Temporal lobectomy	Seizure-free after surgery

Gross total resection should be attempted whenever possible. The appropriate treatment, however, whether lesionectomy alone or lobectomy or amygdalohippocampectomy, must be chosen for every individual patient. Trindade et al. reported the case of a 59-year-old man suffering from a giant epidermoid cyst, presenting with bad smell sensations followed by tonic-clonic convulsions. Their patient remained seizure-free at a one-year follow-up post-lesionectomy [14]. Wong et al. published a case similar to ours. In a patient with a CP angle epidermoid cyst extending to the medial aspect of the left temporal lobe, they found conservative treatment alone to have little impact. Their patient became seizure-free after cytoreductive surgery [15]. Hanft et al. [10] described awake right temporal craniotomy for a right superior temporal lobe epidermoid cyst achieving seizure freedom at five-year follow-up. Our patient’s seizures began with a focal onset in the form of déjà vu experiences, which are typically associated with the mesial portion of the temporal lobe. His feelings of fear may have originated from the abnormal neuronal activity near the amygdala. His postictal prosopagnosia may have been linked to epileptogenic involvement of the fusiform gyrus responsible for face recognition [16,17]. His MRI scan showed no perilesional edema or any sign of parenchymal involvement. We hypothesized that his seizures arose from the mass effect of the lesion. Therefore, we performed a lesionectomy alone and decided to leave a small remnant of the capsule adherent to the mesial temporal lobe to reduce the risk of surgical morbidity. Therefore, we decided to follow up our patient with an MRI after three months to detect early recurrence. Radiation can be considered in a rare case of malignant transformation of the tumor [18,19]. Generally, a thorough histopathological workup is paramount to distinguish this lesion from other neoplasms that are prone to cause temporal lobe epilepsy including gangliogliomas and dysembryoplastic neuroepithelial tumors (DNET).

## Conclusions

Temporal lobe epilepsy is a rare manifestation of epidermoid cysts. No treatment guidelines exist, but surgical management can lead to seizure freedom in most cases. The clinical presentation, imaging studies, and the epileptogenic focus can guide the selection of the appropriate surgical strategy in the individual patient.
